# A methodological framework for road accident severity prediction for indian highways using machine learning models

**DOI:** 10.1016/j.mex.2025.103728

**Published:** 2025-11-19

**Authors:** Humera Khanum, Anshul Garg, Mir Iqbal Faheem, Rushikesh Kulkarni

**Affiliations:** a Civil Engineering, Symbiosis Institute of Technology, Symbiosis Knowledge Village, Near Lupin Research Park, Gram Lavale, Mulshi, Pune, 412115, Maharashtra, India; b School of Civil Engineering, Lovely Professional University, Jalandhar, Delhi-G.T. Road, Pagwara, 144411, Punjab, India; c Civil Engineering, Deccan College of Engineering and Technology, Hyderabad, India

**Keywords:** Road accident severity prediction, Machine learning models, Random forest model, Gradient boosting model, Indian highways

## Abstract

This study introduces a methodological framework for predicting road accident severity using a SHAP-enhanced Machine Learning model. Road traffic accidents remain a major global concern, with India reporting over 150,000 fatalities annually. Traditional models fail to capture the complex relationships among various risk factors. This research applies machine learning, specifically Random Forest and Gradient Boosting, to identify and analyse key factors influencing accident severity. SHAP values are used to enhance model interpretability, providing insights into the contribution of each feature.

• Develop a Random Forest model and a Gradient Boosting model to predict road accident severity based on a comprehensive set of features.
• Utilise SHAP to identify and rank the importance of features, such as vehicle type, weather, and road conditions.
• Model performance is evaluated using accuracy, precision, recall, F1-score, and confusion matrices. Polynomial curve fits are used only as post-hoc visualizations of the Actual–Predicted relationship (on ordinal codes), not as classifier evaluation metrics.
  The findings highlight that factors like vehicle type, accident location, and road conditions significantly influence accident severity. This approach provides a scalable and interpretable framework for improving road safety on Indian highways, offering data-driven insights for proactive safety measures and infrastructure enhancements.

## Graphical abstract

**Figure:**
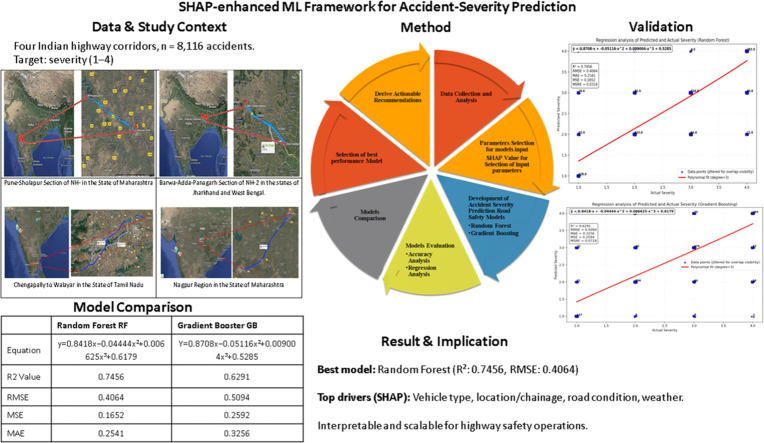

**Table:** 

Specifications table	
**Subject area**	Engineering
**More specific subject area**	Machine Learning, Road Safety, Traffic Engineering
**Name of your method**	Accident Severity Prediction using Machine Learning Random Forest and Gradient Boosting Models
**Name and reference of original method**	Not Applicable
**Resource availability**	The Prediction dataset and Jupiter notebook scripts used in this study are available from the corresponding author E-mail upon reasonable request.

## Background

Road traffic accidents remain a critical global concern, imposing a substantial burden on public health and economic productivity. According to the World Health Organization (WHO), approximately 1.35 million people die in road traffic accidents every year, with many others suffering from long-term disabilities that diminish their quality of life [1]. These tragic outcomes underscore the urgent need for effective intervention strategies and policies to mitigate the severity of road accidents and reduce fatalities.

In India, road traffic accidents account for a significant proportion of global traffic-related deaths, with the country witnessing an alarmingly high number of accidents annually [1]. Despite various road safety initiatives and improvements in infrastructure, the number of accidents continues to rise, demonstrating the need for more predictive and preventive measures. Critical factors such as speeding, impaired driving, poor road conditions, weather hazards, and erratic driver behavior significantly influence accident severity [2].

Traditional statistical models such as linear regression, Poisson regression, and Negative Binomial regression have been extensively utilized in the analysis of road accidents. These approaches predominantly take into account fundamental variables, including spatial location, temporal characteristics, and road classification, to establish systematic relationships with accident occurrence rates [[3], [4], [5]]. Traditional statistical models have helped in understanding road accidents to some extent, but they often struggle when things get complicated. For example, models like linear regression expect a straightforward, one-to-one relationship between factors, which doesn’t always happen in real life. Factors such as speed, weather, and road design don’t just add up, they interact in complex ways that influence how serious an accident turns out to be. Because of this, these simpler models can sometimes miss the real-life scenarios in the data about accidents [6]. The downsides of classical statistical models have led researchers to explore more sophisticated techniques, especially Artificial Intelligence (AI) and Machine Learning (ML). ML methods like Random Forests (RF), Support Vector Machines (SVM), Gradient Boosting (GB), and Neural Networks (NN) excel at tackling big and intricate datasets, making it possible to uncover subtle patterns and complex relationships that straightforward models often overlook. Advanced machine learning approaches can predict road accidents and their severity more accurately by taking into account the complex relationships among factors like road design, traffic conditions, and environment. These methods excel at capturing non-linear connections and subtle dependencies between variables, leading to better prediction quality and deeper understanding of what actually drives accident outcomes. By using ML, researchers and policy makers can identify the most influential factors affecting accident severity and design more effective road safety interventions [6,7].

By integrating ML models with real-time data like weather conditions, traffic flow, and vehicle types, the ability to predict severity of accidents improves significantly [8]**.** This real-time data fusion enables more precise and timely forecasts, allowing for targeted interventions that may prevent accidents before they happen. Such adaptive systems can enhance road safety by providing actionable insights to drivers, traffic managers, and policymakers, eventually helping reduce fatalities and improve traffic management efficiency.

This research aims to contribute to the growing body of knowledge by developing predictive models for road accident severity prediction using machine learning algorithms. By applying advanced methods like RF and GB, this study pursues to identify the key risk factors associated with accident severity and offer actionable recommendations for enhancing road safety, particularly in high-risk areas of Indian Highways.

## Study area

### Description of study area and accident data

Four distinct highway segments under NHAI jurisdiction were chosen to ensure geographic and operational diversity:

1. Pune–Solapur (NH‑9, km 144 400–249 000, Maharashtra): 2804 records
2. Jharkhand & West Bengal Region - Barwa‑Adda–Panagarh (NH‑2, km 398 240–521 120, Jharkhand & West Bengal): 3710 records
3. Tamil Naidu Region - Chengapally–Walayar (Chainages 102 035–144 680 & 170 880–183 010, Tamil Nadu): 422 records
4. Nagpur Region (Maharashtra): 1180 records

The location details of the study areas selected stretches are, shown in Fig. 1a, Fig. 1b, and Fig. 1c.

**Fig. 1a:**
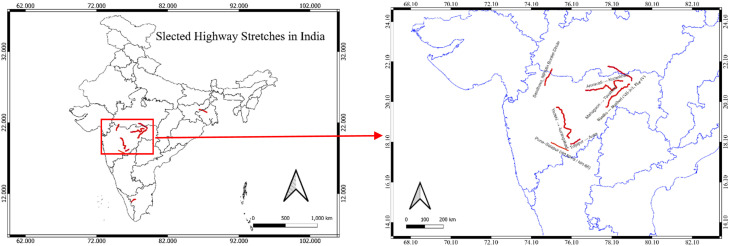
Study Areas comprises of Pune-Solapur and Nagpur Region.

**Fig. 1b:**
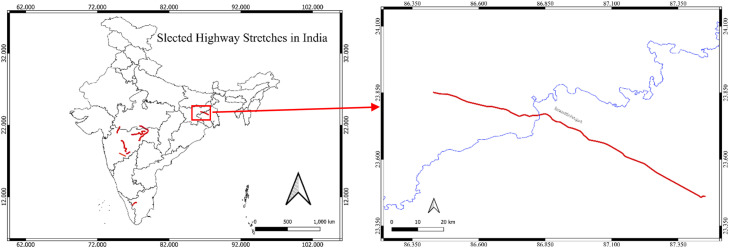
Study Areas comprises of Jharkhand and West Bengal Region.

**Fig. 1c:**
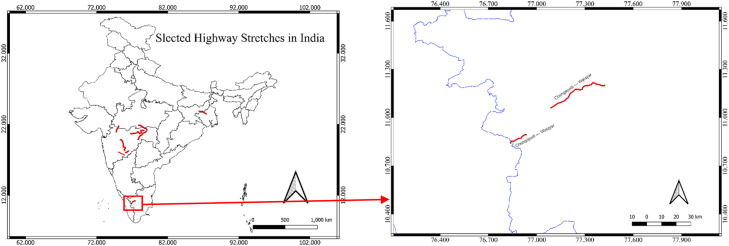
Study Areas comprises of Tamil Nadu Region.

Table 1 presents the comprehensive dataset comprising 8116 accident records from the four highway projects. The dataset's diverse geographic and traffic conditions facilitate a comprehensive analysis of accident severity prediction using RF and GB models, while also allowing for the examination of individual feature significance through Shapley value analysis.

**Table 1:** Summary of accident datasets for the four stretches in India.

Sl. No	Stretch	Accident Data (Nos)
1	Pune-Sholapur Region	2804
2	Jharkhand and West Bengal Region	3710
3	Tamil Nadu Region	422
4	Nagpur Region	1180
	**TOTAL**	**8116**

## Method details

This study offers a key contribution that goes beyond just compiling a new dataset of 8116 accidents from multiple Indian highway corridors. An interpretable modeling framework is introduced that uses SHAP (SHapley Additive Explanations) in two powerful ways: first, to select the most important features before training, and second, to fully explain the model's predictions after they are made. The work then provides a thorough comparison of RF and GB models, detailing their performance using comprehensive, class-specific metrics and a deep analysis of where misclassifications occur. Finally, the findings are benchmarked against other recent studies on accident severity to confirm the model's accuracy and the validity of the interpretability insights. These methodological choices ensure the severity predictions are transparent, reliable, and directly relevant for policy-making, making the framework useful far beyond this specific dataset.

**Fig. 2:**
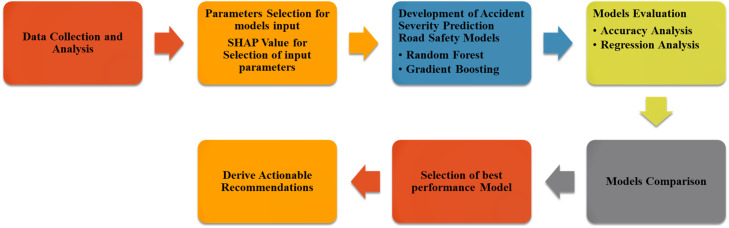
Overall framework of methodology.

The present study employs a structured, multi-stage research design aimed at developing and validating ML-based models for predicting road accident severity on Indian highways. Fig. 2 illustrates the overall framework.

### Data pre-processing

The dataset utilized for this study comprises road accident data collected from selected stretches of highways under the jurisdiction of the National Highways Authority of India (NHAI). The data was provided by the respective Concessionaires and covers four major highway projects across multiple states, representing diverse geographic, traffic, and environmental conditions. The consolidated dataset of 8116 accidents, accessible via Zenodo (https://doi.org/10.5281/zenodo.16946653), provides a robust basis for modeling diverse accident scenarios.

**Fig. 3:**
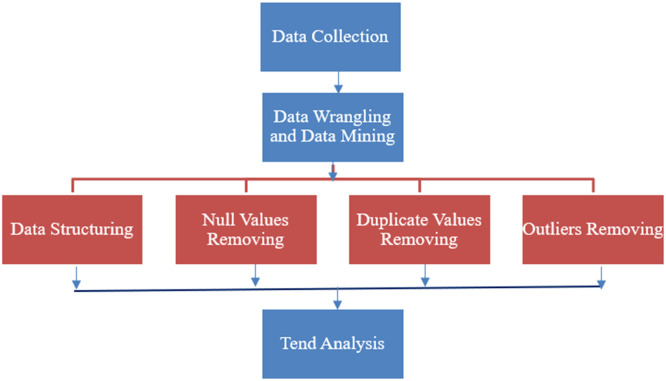
Flowchart for data wrangling and data mining.

Data wrangling and mining techniques (Fig. 3) are used to clean and pre-process the data. Raw accident data often includes missing or inconsistent values, duplicates, and outliers that must be addressed to ensure the dataset's integrity. To clean and pre-process the data, removing inconsistencies, irrelevant information, and other issues that may hinder the analysis process.

Data Structuring is done by formatting the data into a consistent structure (e.g., tables or data frames), ensuring each data entry has critical attributes such as timestamp, location, accident severity, and vehicle type.

Null Value Handling: Identified missing values within the dataset. Remove missing values. For categorical data, replaced missing values with the most frequent category or eliminated the rows with missing values if they were non-critical. Outlier Detection and Removal: Identified outliers in the dataset using statistical techniques such as z-scores or Interquartile Range (IQR). Removed or adjusted extreme values that may distort the overall analysis (e.g., unusually high accident severity scores that are not representative of the majority of the data). Trend Analysis: analyze and identify trends and patterns within the accident data, focusing on factors such as road conditions, weather, and vehicle type, which may influence accident severity. In this step, the primary goal is to recognise correlations between various features and accident severity. Through visualisation, patterns that offer valuable insights into risk factors can be identified.

### Variable selection and encoding

**Fig. 4:**
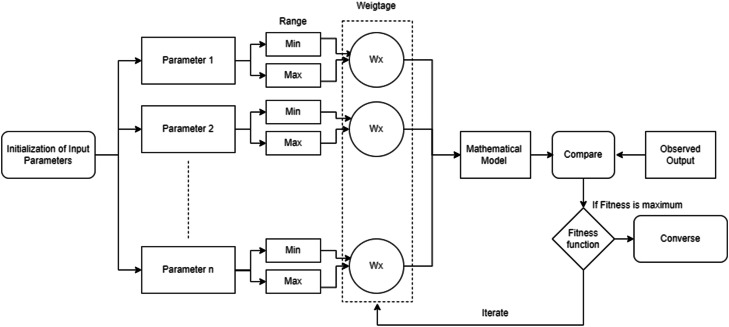
Flowchart for determining influencing parameters.

In this phase of data preprocessing, the relevant variables for predicting accident severity are carefully selected and encoded. The selection process begins with the identification of variables that directly influence accident occurrence. These variables encompass a variety of factors, including road conditions, vehicle type, weather conditions, driver behavior, time of the accident, and location-specific characteristics. To ensure a focused approach, the importance of each variable is assessed based on its relevance to accident severity, helping to retain only those that provide meaningful insights for the prediction model Fig. 4 and Table 2.

**Table 2:** Variables directly involved in accidents.

Sl No	Variables directly involved in accidents	Sl No	Variables directly involved in accidents
1	Date	11	Causes-D4
2	Day of Week	12	Road Feature-E
3	Time of Accident	13	Road Condition-F
4	Accident Location-A	14	Intersection Type-G
5	Accident Location-A Chainage-km	15	Weather Conditions-H
6	Accident Location-A Chainage-km-Road Side	16	Vehicle Type Involved-J-V1
7	Accident Severity -C (Target Variable)	17	Vehicle Type Involved-J-V2
8	Causes-D1	18	Vehicle Type Involved-J-V3
9	Causes-D2	19	Vehicle Type Involved-J-V4
10	Causes-D3		

Once the relevant variables are identified, the next critical step is encoding. The raw accident data typically contains both numerical and categorical data types. For effective use in ML models, it is essential to standardize these variables. Categorical variables, such as road type, weather conditions, and vehicle type, are transformed into numerical representations. This transformation ensures that these variables are compatible with the algorithms used for predictive modeling. The following Table 3 summarizes the key attributes selected from the dataset, along with their respective categories and encodings:

**Table 3:** Dataset attributes and their respective encoding schemes.

Attribute	Description	Encoded Format
**Accident Index**	Unique identifier for each accident	Numeric
**Day of Week**	Day of the week on which the accident occurred	1-Sunday, 2-Monday, 3-Tuesday, 4-Wednesday, 5- Thursday, 6-Friday, 7-Saturday
**Time of Accident**	Time of day the accident occurred	Numeric (24-hour format)
**Accident Location-A**	Location classification	1-Urban, 2-Rural, 3-Unallocated
**Nature of Accident**	Type of accident (e.g., collision, rollover)	1-Overturning, 2-Head on collision, 3-Rear End Collision, 4-Collision Brush/Side Wipe, 5-Right Turn Collision, 6- Skidding, 7a-Others-Hit Cyclist, 7b-Others-Hit Pedestrian, 7c-Others-Hit Parked Vehicle, 7d-Others-Hit Fixed Object, 7e-Others-Wrong Side Driving, 7f-Others-Hit Animal, 7 *g*- Others-Hit Two Wheeler, 7h-Others-Unknown, 7i-Others- Fallen down, 8-Overtaking vehicle, 9-Left Turn Collision
**Accident Severity-C**	Severity level of the accident	1-Fatal, 2-Grevious Injury, 3-Minor Injury, 4-No Injury
**Road Feature-E**	Road feature (e.g., single lane, dual lane)	1-Single lane, 2-Two lanes, 3-Three lanes or more without a central divider median, 4-Four lanes or more with a central divider along with carriageway width
**Weather Conditions-H**	Weather at the time of the accident	1-Fine, 2-Mist/Fog, 3-Cloud, 4-Light Rain, 5-Heavy Rain, 6-Hail/sleet, 7- Snow, 8-Strong Wind, 9-Dust Storm, 10-Very Hot, 11-Very Cold, 12-Other extraordinary weather conditions
**Vehicle Type-J**	Type of vehicle involved	1-Car/Jeep/Van, 2-SUV, 3-Bus, 4-Mini Bus, 5-Truck, 6- Two Wheeler, 7-Three Wheeler, 8-Cycle, 9-Pedestrian, 10- Tractor, 11-Unknown, 12-Animal, 13-Objects, 14-LCV, 15- MAV
**Road Condition-F**	Condition of the road (e.g., straight, curve)	1-Straight Road, 2-Slight Curve, 3-Sharp Curve, 4-Flat Road, 5-Gentle incline, 6-Steep incline, 7-Hump, 8-Dip

Inputs were consistently encoded prior to modeling. Categorical variables were cleaned and label-encoded; numeric variables were left on their native scale. No feature standardization or normalization (e.g., StandardScaler, MinMaxScaler) was applied, as tree-ensemble learners are not sensitive to feature scaling.

The next critical step involves the application of SHAP (Shapley Additive Explanations) values for identifying the most influential parameters in the prediction of accident severity. By using SHAP values, the model's interpretability is enhanced, and key features that drive accident severity predictions can be determined. This process ensures that the final selection of input parameters is both effective and aligned with the goal of accurate prediction.

This can be applied as follows: Calculate SHAP values for each parameter (e.g., weather, vehicle type, etc.). Identify influential parameters: The SHAP values will indicate the contribution of each parameter to the model’s predictions. Higher absolute SHAP values mean the parameter had a larger influence. Weightage Assignment: The SHAP values can be used to assign weightage (Wx) to the parameters. Parameters with higher SHAP values can be given higher weightage in the next iteration. Optimization: The iterative process in the flowchart can be guided by SHAP values, which can help determine which parameters are most important for improving the model's performance (Fig. 3).

### SHAP-based feature finalization

**Fig. 5:**
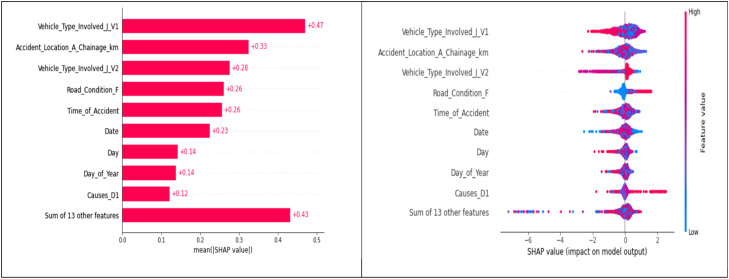
SHAP value analysis.

Feature importance was quantified using mean absolute SHAP values computed on the training data within stratified k-folds. Per feature, SHAP values were averaged across folds to obtain a stable ranking. The top-N features (*N* = 12) were retained for modeling; additionally, domain-critical environmental variables (e.g., Weather Conditions-H) were retained when near the cut-off to preserve known exposure effects. The target (Accident_Severity_C) was excluded from the input set during training/inference. (See Fig. 5 and Table 4.)

**Table 4:** Final variables after applying SHAP-VALUE analysis for modelling.

Sl No	Final Variables after applying SHAP-VALUE Analysis for modelling	Sl No	Final Variables after applying SHAP-VALUE Analysis for modelling
1	Date	8	Causes-D1
2	Day of Week	9	Road Feature-E
3	Time of Accident	10	Road Condition-F
4	Accident Location-A	11	Weather Conditions-H
5	Accident Location-A Chainage-km	12	Vehicle Type Involved-J-V1
6	Accident Location-A Chainage-km-Road Side	13	Vehicle Type Involved-J-V2
7	**Accident Severity -C (Target Variable)**		

This ensures that the models are trained on the most important and impactful features, enhancing their predictive accuracy and overall performance. By carefully selecting and encoding the variables, this phase of the methodology sets a solid foundation for the subsequent model development and evaluation.

### Model development, model training and validation techniques

**Fig. 6:**
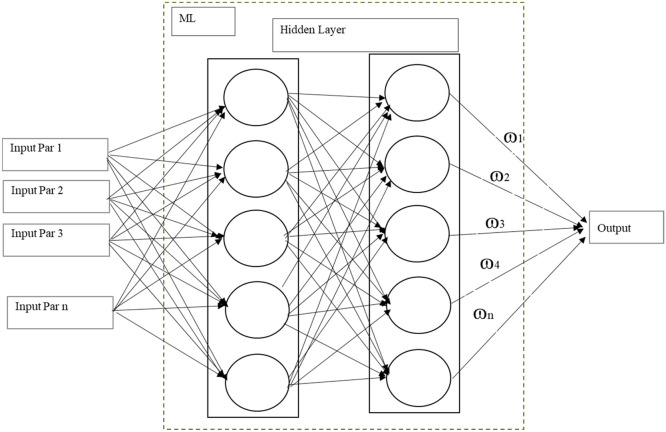
Algorithm machine learning based framework for accident severity prediction road safety models.

**Fig. 7:**
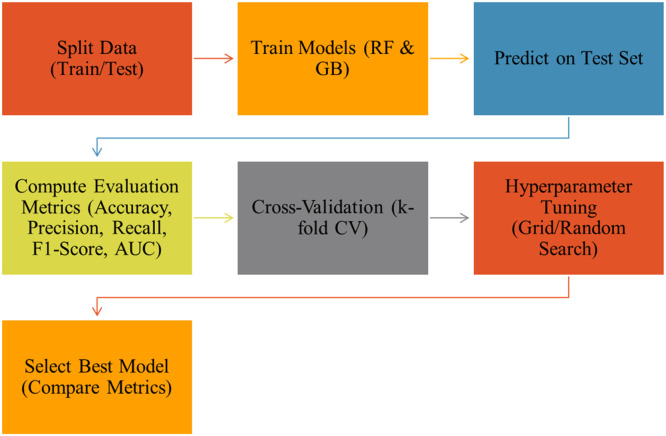
Algorithm for model evaluation and selection of the best performing model.

The Accident Severity Prediction Road Safety Model will be developed in line with the framework using ML, as shown in Fig. 6 and evaluated as per the framework given in Fig. 7:

### Random forest

The RF algorithm is an ensemble learning method used for classification and regression tasks. In this study, RF is applied to predict road accident severity based on the selected variables. The RF model works by constructing multiple decision trees during training and outputting the mode of the classes (classification) or the average prediction (regression) of the individual trees.

Key Steps in RF Development:

The accident severity was modelled as a categorical outcome and a RF classifier using a curated highway accident dataset containing variables (Date, Day_of_Week, Time_of_Accident), locational (Accident_Location_A, chainage and roadside), roadway (Road_Feature_E, Road_Condition_F), environmental (Weather_Conditions_H), and vehicle descriptors (Vehicle_Type_Involved_J_V1, Vehicle_Type_Involved_J_V2). After loading the CSV with explicit column names, the target variable (Accident_Severity_C) is separated from the predictors. All object-type predictors are converted to numeric codes via per-column LabelEncoder to allow tree-based learning while preserving category identities; infinite values are coerced to missing, and observations with missing values are removed. The data are randomly split into 75 % training and 25 % testing with a fixed seed (random_state=42) for reproducibility. After instantiating a RandomForestClassifier (*n_estimators* = 5000, *max_depth* = 22), the model was fitted to the training set. Predictive performance is evaluated on the held-out test set using overall accuracy and the classification report (class-wise precision, recall, and F1-score, plus macro/weighted averages) together with a confusion matrix to analyse error distribution across severity levels. For interpretability and auditability, test-set results were compiled in the table that joins each case’s original (de-encoded) features with its Actual and Predicted severity, and export this to an Excel file to support downstream error analysis, slice-wise diagnostics, and qualitative review**.** Impurity-based feature importance plot from the fitted model was extracted and visualized to identify the most influential predictors.

### Gradient boosting

GB is another powerful ensemble method that builds models sequentially, with each model correcting the errors of its predecessor. Unlike RF, which builds independent trees, GF constructs trees in a manner where each subsequent tree attempts to reduce the residual errors of the combined previous trees.

Key Steps in GB Development:

The accident severity was modelled using GB as a machine learning technique to predict accident severity based on a variety of factors. The dataset from File.csv, consists of multiple attributes such as temporal data (Date, Day_of_Week, Time_of_Accident), location (Accident_Location_A, chainage, roadside), road characteristics (Road_Feature_E, Road_Condition_F), environmental conditions (Weather_Conditions_H), and vehicle-related information (Vehicle_Type_Involved_J_V1, Vehicle_Type_Involved_J_V2). The same unified preprocessing as for RF was applied: infinite values were converted to NaNs; occasional categorical gaps were imputed with the mode; any remaining incomplete records were removed; categorical features were label-encoded; *Accident_Severity_C* was isolated as the target; and the dataset was split into 75 % training and 25 % testing with a fixed random seed (*random_state* = 42) for reproducibility.

A GradientBoostingClassifier was instantiated with 5000 estimators and a maximum depth of 22 to model non-linear relationships and capture complex interactions between predictors. The model was then trained on the training set and evaluated on the test set. Performance was assessed using accuracy, classification report (including precision, recall, F1-score, and macro/weighted averages), and the confusion matrix to assess the distribution of errors across severity classes. Feature importance were extracted from the fitted model and visualized as a horizontal bar plot to highlight the most influential predictors. The results from this analysis, including the accuracy score, classification report, confusion matrix, and feature importance plot, were saved and used for further investigation and model interpretation.

Note: Models were trained without feature scaling; inputs used label-encoded categorical variables and native-scale numeric variables

### Hyperparameter tuning

A two-stage procedure was used. Stage-1 (Randomized search): stratified 10-fold CV with RandomizedSearchCV explored broad ranges—RF: n_estimators ∈ {500, 1000, 2000, 3000, 5000}, max_depth ∈ {None, 10, 16, 22, 30}, min_samples_split ∈ {2, 5, 10}, min_samples_leaf ∈ {1, 2, 4}, max_features ∈ {“sqrt”, 0.5, None}; GB: n_estimators ∈ {500, 1000, 2000, 5000}, learning_rate ∈ {0.01, 0.05, 0.1}, max_depth ∈ {6, 10, 16, 22}, subsample ∈ {0.6, 0.8, 1.0}, min_samples_split ∈ {2, 5, 10}, min_samples_leaf ∈ {1, 2, 4}. Stage-2 (Refinement grid): a compact GridSearchCV centered on the top-3 candidates per model. The primary metric was macro-F1 (class imbalance aware), with accuracy used as a tie-breaker and the one-SE rule favoring the simpler model when scores were statistically indistinguishable. All searches used fixed seeds (42) and identical folds. Final selections were RF: n_estimators = 5000, max_depth = 22, (min_samples_split = 2, min_samples_leaf = 1, max_features = “sqrt”); GB: n_estimators = 5000, learning_rate = 0.1, max_depth = 22, subsample = 1.0 (other parameters at defaults).

## Performance metrics and evaluation criteria

### Post-hoc polynomial curve fit (Visualization only)

Polynomial curve fitting was used solely as a post-hoc visualization to illustrate the non-linear alignment between Actual and Predicted severity (ordinal codes). Reported R²/MAE/RMSE values describe the quality of this trend depiction; they were not used to train, tune, or select classifiers. All model selection and comparisons rely on classification metrics (accuracy, macro-F1, precision, recall) and confusion matrices.

### Comparison between RF and GB

The performance of the RF and GB models in predicting accident severity was systematically compared. Both models were evaluated based on their prediction output, established performance metrics (such as precision, recall, and F1-score), and computational efficiency to identify the superior model for accident severity prediction. This comparison aimed to determine which model is more accurate, reliable, and interpretable within the context of the accident dataset utilized in this study.

### Rationale for model selection

RF and GB were prioritized because the task uses heterogeneous, tabular inputs (temporal, location, roadway, environment, vehicle) where tree ensembles are known to model non-linear effects and interactions effectively while remaining inspection-friendly via importance plots and SHAP explanations. On this dataset, RF achieved the highest test performance and was selected as the working model, with GB as a competitive baseline; detailed metrics and the model-selection table are provided in Results. External evidence summarized in the paper further indicates that RF variants compare favorably with SVM and neural-network baselines on similar accident-severity problems, supporting this choice of primary learners.

## Method validation

### K-Fold cross-validation

To ensure a robust and generalized evaluation of the proposed models and to mitigate the risk of overfitting, K-fold cross-validation was systematically incorporated into the model training and selection phase. This technique is crucial as it utilizes the entire dataset for both training and validation, ensuring that the model's performance metrics are not dependent on a specific arbitrary partition and providing a more reliable estimate of its true generalization capability. Various values for K were rigorously tested, and the 10-fold cross-validation (*K* = 10) configuration was selected. This choice provided the best balance of variance and bias, consistently yielding the most stable and superior performance metrics across all evaluated models, thereby confirming the reliability of the validation results reported in this study.

Hyperparameters were selected via a stratified 10-fold randomized-plus-grid search optimizing macro-F1; the procedure consistently ranked RF ≥ GB. The chosen configurations (RF: n_estimators = 5000, max_depth = 22; GB: n_estimators = 5000, learning_rate = 0.1, max_depth = 22) delivered the test results reported above.

### Accident severity prediction model output and validation using RF

**Fig. 8:**
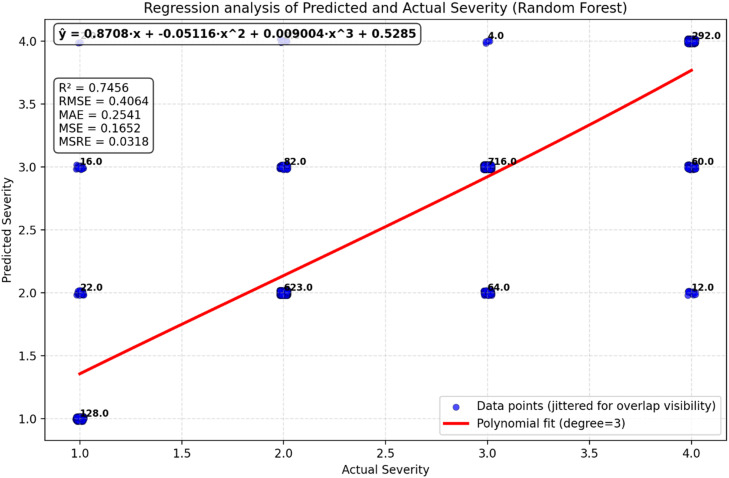
RF model validation polynomial regression.
• The scatter of points and the annotations on overlapping points highlight the concentration of similar data points in the plot, which is an important observation in regression analysis. The heat map represents the detailed distribution (Fig. 9).

• The model correctly predicts accident severity in 86.65 % of cases, indicating high overall accuracy.
• The model showed high precision and low recall for class 1.
• Balanced performance for Classes 2, 3, and 4, with high precision and recall.
• Misclassification patterns suggest areas for model improvement, particularly in distinguishing between Classes 2 and 3.
• The polynomial regression curve *y* = 0.8418x−0.04444×^2^+0.006625×^3^+0.6179 provides a good fit for the data, as evidenced by the reasonably high R² value (0.7456). The polynomial curve adequately captures the non-linear relationship between Actual and Predicted Severity (Fig. 8). These curves visualize global monotonic alignment only; model performance and selection are based on classification metrics reported above.

**Fig. 9:**
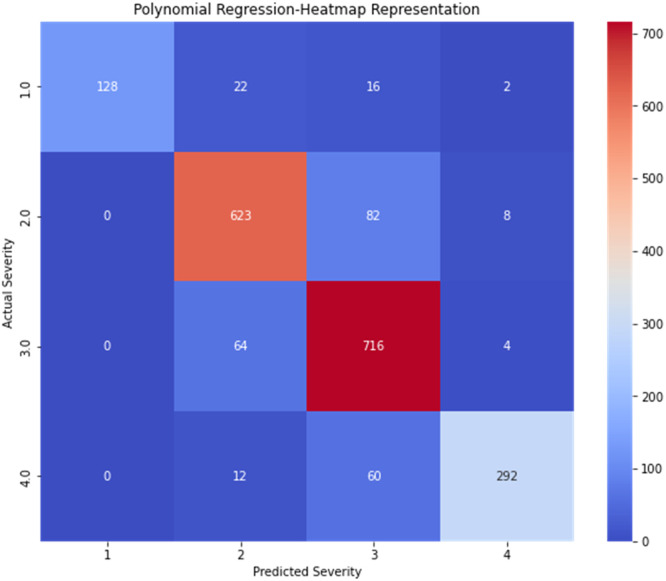
RF model validation heatmap representation.
• Year-wise alignment between observed and RF-predicted severity further supports temporal robustness, with only minor deviations across 2013–2023 (Fig. 10).

**Fig. 10:**
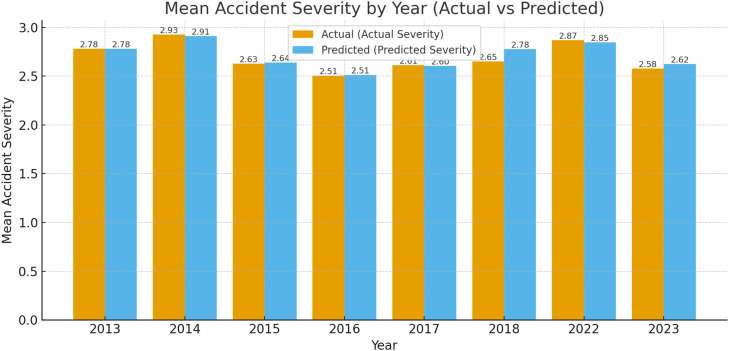
Comparison of actual and RF predicted accident severity by year (2013–2023).
• This also suggests that the model has learned the underlying patterns from the dataset but may have more accuracy in areas with fewer overlapping Points.

### Accident severity prediction model output and validation using GB

**Fig. 11:**
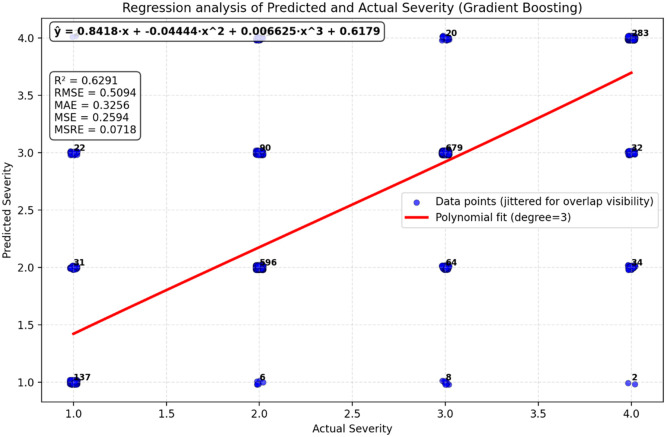
GB model validation polynomial regression.
• The jittered scatter plot reveals how densely the data points are distributed, particularly in areas with many overlapping values. The heat map represents the detailed distribution (Fig. 12),

• The model correctly predicts accident severity 83.28 % of the time, indicating high overall accuracy.
• Class 1 (Fatal) has high precision but lower recall, indicating a need for better identification of fatal accidents.
• Balanced performance for Classes 2, 3, and 4 with good precision and recall.
• Misclassification patterns suggest areas for model improvement, particularly in distinguishing between Classes 2 and 3.
• The polynomial regression curve *Y* = 0.8708x−0.05116×^2^+0.009004×^3^+0.5285 fits the data reasonably well, capturing the relationship between Actual Severity and Predicted Severity with a moderate R² value of 0.6291 (Fig. 11). These curves visualize global monotonic alignment only; model performance and selection are based on classification metrics reported above.

**Fig. 12:**
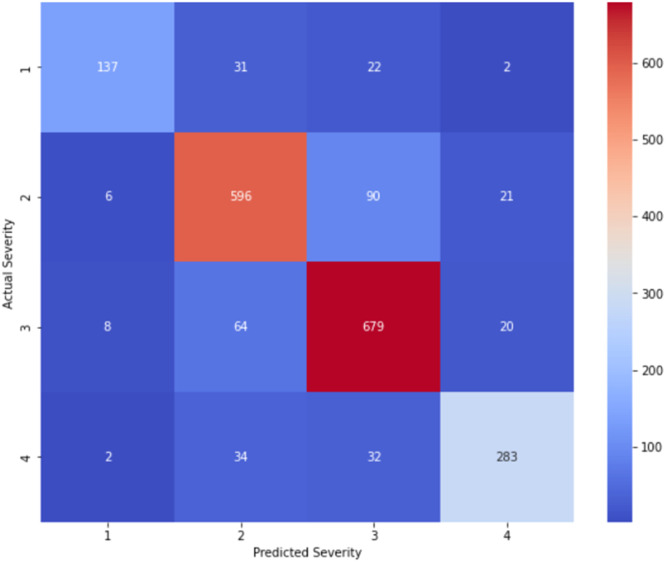
GB model validation heatmap representation.
• The year-wise comparison indicates broadly similar trends but with slightly larger gaps than RF, consistent with GB’s lower test performance (Fig. 13)

**Fig. 13:**
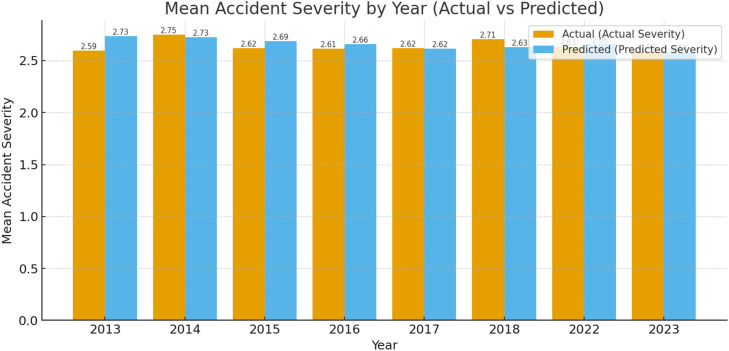
Comparison of actual and GB predicted accident severity by year (2013–2023).
• Metrics like R², RMSE, and MAE show that the polynomial regression model performs fairly well, but there is still room for improvement. For example, the RMSE and MAE values suggest that some predictions deviate significantly from the actual values, particularly in areas where the data points are more clustered.

### Model comparison and best model selection

After evaluating and validating the models it can be stated that RF clearly outperforms GB across all major regression metrics (R², RMSE, MAE, MSE, MSRE) Table 5. This suggests that RF is a better model for predicting Predicted Severity from Actual Severity in this dataset. GB performs well but shows slightly higher errors and less explanatory power, making it less suitable for this specific dataset in comparison.

**Table 5:** RF and GB polynomial regression analysis for validation and comparison.

	RF	GB
Equation	*y* = 0.8418x−0.04444×^2^+0.006625×^3^+0.6179	*Y* = 0.8708x−0.05116×^2^+0.009004×^3^+0.5285
R^2^ Value	0.7456	0.6291
RMSE	0.4064	0.5094
MSE	0.1652	0.2592
MAE	0.2541	0.3256

Hence, the best-performing model, RF, is selected for accident severity prediction. The chosen model will be used for further analysis and to provide actionable recommendations for improving road safety interventions.

### External benchmarking and comparative results

To contextualize the model’s performance, results are compared with two recent, peer-reviewed studies that also predict traffic accident severity using random-forest–based approaches on independent datasets from the United States [9] and China [10]. Because datasets, class definitions, class balance, and validation protocols differ, strict like-for-like numerical comparison is not appropriate; however, a side-by-side synthesis clarifies where the present findings align with or exceed established baselines and how tuning and interpretability choices (e.g., SHAP versus feature importance/partial dependence) influence reported metrics. The following paragraph summarizes datasets, evaluation setups, headline metrics, and interpretability tools across the three studies.

Across three independent datasets, random-forest–based models consistently dominate results. In our Indian-highway dataset (8116 accidents), the RF model attains 86.65 % test accuracy (GB: 83.28 %), establishing the highest performance on this data. In Montgomery County (USA) [9], a Bayesian-optimized RF (BO-RF) yields macro-F1 = 0.57 and AUC = 0.9625 (baseline RF: *P* = 0.70, F1 = 0.54, AUC = 0.958), with superiority confirmed under 10-fold CV and robust to 70/30 and 60/40 splits.

On the Chinese NAIS data [10], RF outperforms RBF-NN, BP-NN, and SVM, reaching Accuracy = 80 %, Recall = 0.83, F1 = 0.82, ROC = 0.85, using a train–test setup where 2240 of 2800 records are used for training. While absolute numbers are not directly comparable due to differing class balances and validation designs, all three studies converge on RF variants as state-of-the-art for severity prediction; notably, BO-tuning boosts AUC/macro-F1 in the US case, and SHAP-guided selection in our study strengthens accuracy and interpretability on Indian highways.

### General applicability of the model

This study significantly extends prior research by introducing a comprehensive methodological framework that combines advanced ML techniques with the SHAP methodology. While existing literature often focuses solely on predictive accuracy, this framework emphasizes interpretability and actionable insight alongside performance, providing a deeper understanding of the complex feature interactions driving accident severity, particularly within the challenging context of Indian highways. Crucially, the proposed ML-SHAP methodological framework is transferable; although the current dataset focuses on Indian infrastructure, the overall approach can be readily applied to road safety data from any geographical location, provided equivalent traffic, road geometry, and demographic data are available. This enhances the generalizability of this technique for global road safety prediction efforts.

## Conclusions

This study developed and evaluated accident-severity prediction models for Indian national highways using supervised learning, with RF as the best-performing model (test accuracy = 86.65 %) and GB as a competitive baseline (83.28 %). The analysis documents class-wise behavior and visually explains residual structure via overlap-aware scatter/heatmap views, highlighting confusion between adjacent classes that informs thresholding and sampling decisions in practice. Beyond accuracy, a primary contribution is interpretability. The pipeline couples model performance with factor salience (via Shapley-style analyses) to indicate which roadway, temporal, and contextual attributes most influence severity, information that can directly guide engineering countermeasures and operational prioritization on similar corridors. Comparative synthesis against peer-reviewed RF-based studies on U.S. and Chinese datasets shows consistent strength of RF variants while underscoring dataset and protocol specific differences, situating the obtained results within the international literature. Collectively, these findings provide a reproducible, interpretable baseline for highway safety planning on Indian corridors and a foundation for future extensions.

## Limitations

Despite the model's strong performance, the current study has certain limitations. First, the evaluation relies on retrospective records (8116 accidents) from only four Indian highway corridors, which limits its immediate transferability to other geographic regions. Second, the performance was assessed using a single internal train-test split, meaning out-of-sample generalization to different road networks globally or time periods remains to be formally confirmed. Future work will focus on external validation across diverse regions, incorporating additional exposure variables, and establishing a periodic model recalibration protocol to manage data drift over time.

## Ethics statements

This study utilized accident data received on request by the authors from the concessioners and safety audit consultants of the respective study areas under the National Highways Authority of India.

## CRediT authorship contribution statement

**Humera Khanum:** Conceptualization, Data curation, Methodology, Validation, Writing – original draft, Writing – review & editing. **Anshul Garg:** Conceptualization, Supervision, Writing – review & editing. **Mir Iqbal Faheem:** Conceptualization, Supervision, Writing – review & editing. **Rushikesh Kulkarni:** Software, Validation, Writing – review & editing.

## Declaration of competing interest

The authors declare that they have no known competing financial interests or personal relationships that could have appeared to influence the work reported in this paper.

## Data Availability

I have shared the link for accident data in the manuscript and The Prediction dataset and Jupiter notebook scripts used in this study are available from the corresponding author E-mail upon request.
